# The Role of Lipids in Human Milk and Infant Formulae

**DOI:** 10.3390/nu10050567

**Published:** 2018-05-04

**Authors:** Alessandra Mazzocchi, Veronica D’Oria, Valentina De Cosmi, Silvia Bettocchi, Gregorio Paolo Milani, Marco Silano, Carlo Agostoni

**Affiliations:** 1 Pediatric Intermediate Care Unit, Fondazione IRCCS Ospedale Ca’ Granda-Ospedale Maggiore Policlinico, Department of Clinical Sciences and Community Health, University of Milan, 20122 Milan, Italy; valentina.decosmi@unimi.it (V.D.C.); carlo.agostoni@unimi.it (C.A.); 2 Pediatric Intensive Care Unit, Fondazione IRCCS Ca’ Granda, Ospedale Maggiore Policlinico, Department of Anesthesia and Intensive Care and Emergency, 20122 Milan, Italy; veronica.doria.vd@gmail.com; 3 Department of Clinical Sciences and Community Health, University of Milan, Branch of Medical Statistics, Biometry, and Epidemiology “G. A. Maccacaro”, 20122 Milan, Italy; 4 Institute of Microbiology Catholic University of the Sacred Heart, 29100 Piacenza, Italy; silvia.bettocchi@unicatt.it; 5 Pediatric Unit, Fondazione IRCCS Ca’ Granda Ospedale Maggiore Policlinico, 20122 Milan, Italy; gregorio.milani@unimi.it; 6 Department of Food Safety, Unit of Human Nutrition and Health, Nutrition and Veterinary Public Health, Istituto Superiore di Sanità, 00161 Rome, Italy; marco.silano@iss.it; 7 SIGENP (Italian Society of Pediatric Gastroenterology, Hepatology, and Nutrition), via Libero Temolo 4 (Torre U8), 20126 Milan, Italy.

**Keywords:** fatty acids, infant formulae, preterm infants, short bowel disease, atopy

## Abstract

The quantity and quality of dietary lipids in infant formulae have a significant impact on health outcomes, especially when fat storing and/or absorption are limited (e.g., preterm birth and short bowel disease) or when fat byproducts may help to prevent some pathologies (e.g., atopy). The lipid composition of infant formulae varies according to the different fat sources used, and the potential biological effects are related to the variety of saturated and unsaturated fatty acids. For example, since lipids are the main source of energy when the normal absorptive capacity of the digestive tract is compromised, medium-chain saturated fatty acids might cover this requirement. Instead, ruminant-derived *trans* fatty acids and metabolites of n-3 long-chain polyunsaturated fatty acids with their anti-inflammatory properties can modulate immune function. Furthermore, dietary fats may influence the nutrient profile of formulae, improving the acceptance of these products and the compliance with dietary schedules.

## 1. Introduction

Dietary lipids are the main source of energy in infants: they account for 45–55% of the total energy provided by human milk and formula in the first six months of life [1]. Furthermore, they are involved in the regulation of cell functions, in inter- and intracellular communication, and in the epigenetic modulation of the genome [2]. The anatomical and functional development of the nervous system seems particularly to depend on the direct supply of DHA and also ARA to some extent (not yet defined) [1,3].

Consequently, the quality of dietary lipids in infant formulae has a significant impact on health outcomes. In infants with limited stores of fats (such as preterms) and/or limitations on fat absorption (such as infants undergoing bowel resection), both the quantity and quality of supplied fats may become more critical, considering the whole nutritional balance. The same considerations apply to disorders where proinflammatory byproducts of some fatty acids have a proven pathogenic role (such is the case with atopy). Therefore, a reappraisal of function and roles of different fat families is relevant to derive indications for formulae fat composition, not just for healthy growing infants, but infants with some specific disorders, to support either normal development while improving the basal, non-physiologic condition.

## 2. Lipid Quality and Role in Human Milk and Infant Formulae

**Figure 1:**
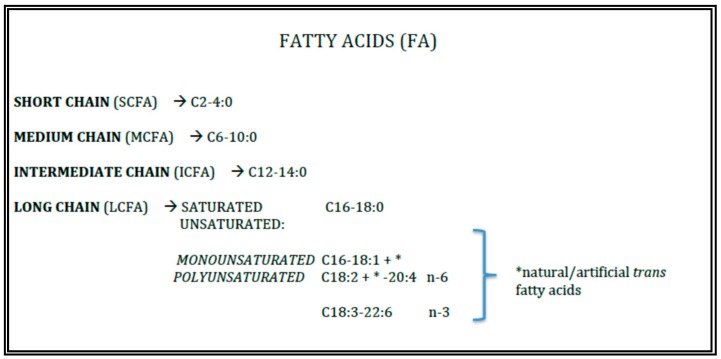
Lipid quality.

Triacylglycerols (TAGs) represent the 98–99% of total fats of human milk and infant formulae. Their properties are devoted from the length and the insaturation grade of the fatty acids (FAs) esterified to the glycerol backbone (Figure 1).

While in human milk the FA composition is dynamic and modulated by maternal diet, in formulae the lipid content is fixed and derived from the vegetable oils [4], fish oils, algae oils, fractionated lipids, and egg phospholipids used as ingredients. The use of dairy fats in infant formulae, even if it has generally decreased, is still popular in some parts of the world [4] and has recently been given attention [5] for some favorable metabolic effects.

### 2.1. Saturated and Monounsaturated Fatty Acids

Saturated fatty acids (SFAs) are abundant in human milk, making up a remarkable 10–12% of the total dietary energy provided by palmitic acid (16:0) [2]. About 70% of the palmitic acid in human milk is esterified to the sn-2 position of TAGs and, since the human lipases hydrolyses dietary TAGs at the sn-1,3 position, palmitic acid is mainly absorbed as glycerol–palmitate [6]. This consideration is important for infant formulae whose lipids are from palm oil, where palmitic oil, once hydrolyzed in bowel lumen, tends to generate insoluble calcium soaps and negatively influences early bone accretion [6].

Human milk contains small amounts of short-chain SFAs (SCFAs, with a carbon chain length < 6C), and 8–10% of medium-chain SFAs (MCFAs, usually with a carbon length of 6–10C) [6]. TAGs containing SCFAs, MCFAs, and to some extent also lauric acid (12C), are more rapidly hydrolyzed by gastrointestinal lipases without the need for bile salts, and their products are more easily absorbed and taken directly to the liver via the portal vein. The ingestion of these FAs, therefore, could provide some benefits under conditions where fat absorption is a limiting factor to reach adequate energy supply [7,8,9,10]. Accordingly, in preterm infants, because of intestinal immaturity, facilitation of fat absorption through the inclusion of MCFAs in the diet might be a promising approach. Yet, there is no demonstrated benefit for energy balance or growth in these patients. On the other hand, the addition of dietary medium-chain triglycerides (MCTs) has been shown to be beneficial in children with severe fat malabsorption such as short bowel syndrome, cystic fibrosis, or severe cholestatic liver disease [8].

Monounsaturated fatty acids (MUFAs) are, along with SFAs, the most represented fatty acid family in human milk and infant formulae, and among them, oleic acid (OA, 18C:1 n-9) is by far the most abundant. In the breastmilk of mothers from Mediterranean countries, where consumption of olive oil is high, OA may reach levels higher than 40%, and total monounsaturated FA higher than 45%, respectively, of total FA in human milk [6].

The longest SFA represent the circulating pool for oxidation. Stearic acid (C18:0) may rapidly be converted to OA, and this pathway indicates the strict metabolic interrelationships between the SFA and the MUFA pool, respectively.

### 2.2. Polyunsaturated Fatty Acids

**Figure 2:**
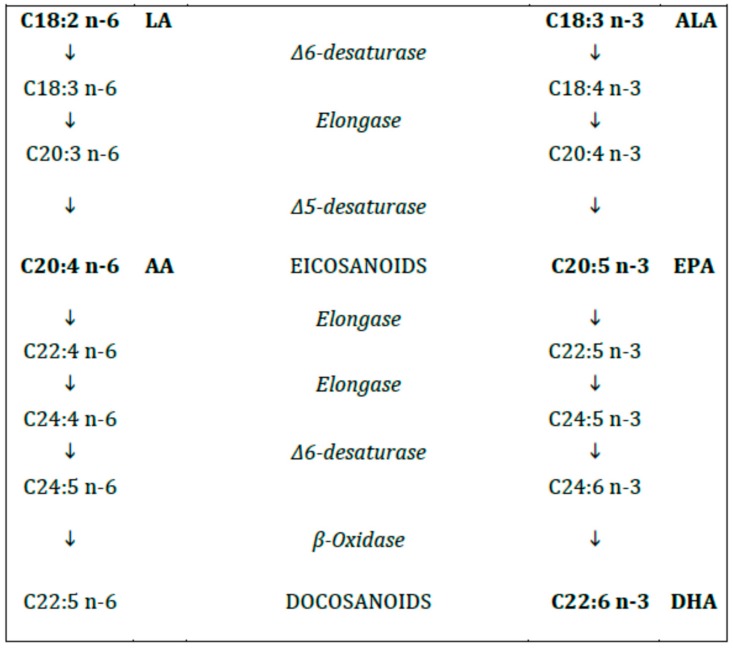
Polyunsaturated fatty acids (n-6 and n-3).

The human enzymatic machinery is not able to endogenously introduce a double bond in the FA molecule at position n-6 and n-3. Therefore, the n-6 and n-3 FAs (6C and 3C, respectively, from the methyl terminal), progenitors linoleic acid (LA, C18:2 n-6) and alpha-linolenic acid (ALA, C18:3 n-3), respectively, must be provided with the diet and are therefore named essential fatty acids (EFAs). Both LA and ALA are metabolized by the same desaturases and elongases to longer and, more unsaturated FAs (Figure 2). The n-6 FA cannot be converted to n-3 FA and vice versa.

In case of deficiency of the EFAs the same enzyme system starts to synthetize a non-essential 20C polyunsaturated fatty acid (PUFA) from MUFA (eicosatrienoic acid, ETE, C20:3 n-9), not relevant from the metabolic standpoint, whose levels are the major indicator of EFA deficiency [11]. 

The long-chain (LC) PUFAs (with chain length > 18C) include γ linolenic acid (GLA, C18:3 n-6), dihomogamma linolenic acid (DHGLA, C20:3, n-6) and arachidonic acid (ARA, C20:4, n-6) for the n-6 series and eicosapentaenoic acid (EPA, C20:5 n-3) and docosahexaenoic acid (DHA, C22:6 n-3) for the n-3 series, respectively [12].

For the abovementioned reasons, the PUFAs composition of human milk primarily, but not only, depends on the mother’s diet. As a matter of fact, breastmilk from vegans shows a high content of LA and ALA, while that from mothers with a high dietary intake of fish has a high content of EPA and DHA [6]. The circulating levels of DHA have been associated to the neurological, visual and IQ performance [7]. The concentrations of DHA in breastmilk are also influenced by polymorphisms in the fatty acid desaturase (FADS) gene cluster [7]. It has to be mentioned that a small amount of PUFA is endogenously synthetized. This amount varies in people with common or uncommon variants of the genes for these enzymes [4].

In infant formulae, LC-PUFAs are provided by adding fish oil, DHA-rich algal oil from *Crypthecodinium cohnii* or ARA-rich fungal oil from *Mortierella alpina*, and phospholipids (lecithin/phosphatidylcholine) from egg yolk [7].

Some of the most potent effects of PUFAs are related to their enzymatic conversion into a series of oxygenated metabolites termed eicosanoids, so called because their precursors are PUFAs with chain lengths of 20 carbon units. Eicosanoids modulate cardiovascular, pulmonary, immune, reproductive, and secretory functions through organ systems in the human body. Eicosanoids are essential for all the functions of human life and include prostaglandins (PG), thromboxanes (TXA), and leukotriene (LT) from DHGLA, ARA, and EPA via cyclooxygenase and lipoxygenase pathways [13].

According to most epidemiological studies, an excess of dietary LA and/or ARA within the n-6 series compared to n-3 parental or derivate byproducts may support stronger proinflammatory and allergic reactions due to the biological differences among n-6 and n-3 derived eicosanoids, respectively. Therefore, as for the case of formulae to be used in preterm infants or those with small bowel syndrome, the ratio n3/n6 LCPUFA should be closer to that of breastmilk with respect to standard formula [8,10].

### 2.3. Trans Fatty Acids

*Trans* fatty acids (TFAs) include unsaturated and polyunsaturated fatty acids with at least one double bond in trans configuration [14].

**Figure 3:**
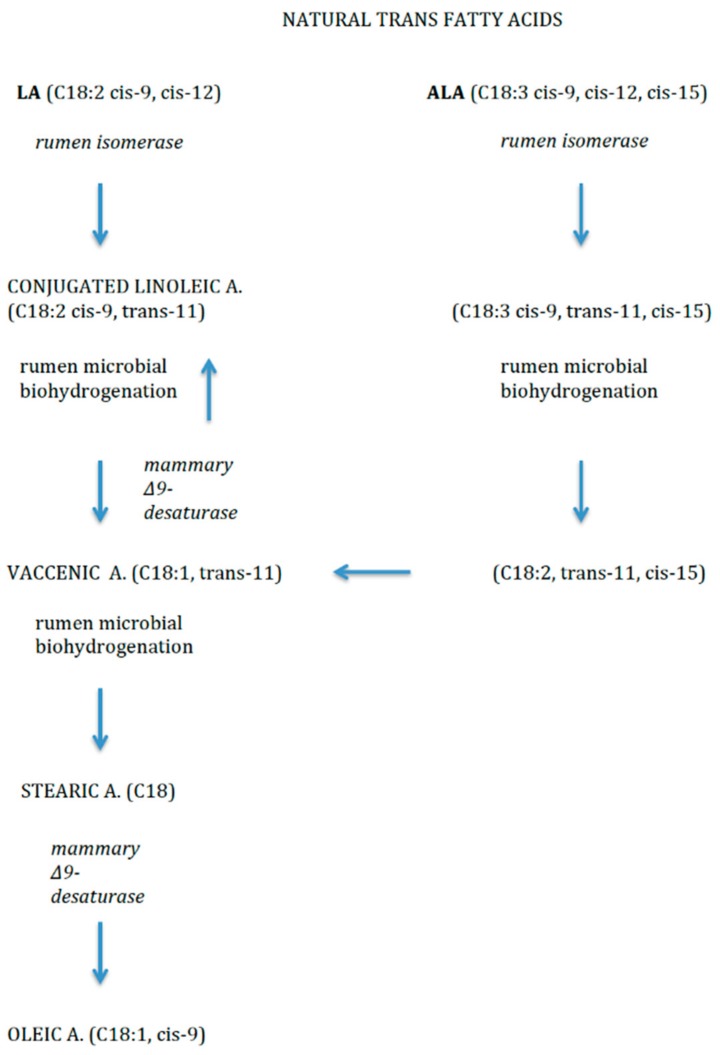
Natural *trans* fatty acids.

Dietary TFAs may come from two sources. Firstly, non-natural source includes the industrial production, by means of partial hydrogenation of vegetable oils (e.g., elaidic acid, C18:1, *trans*-9). Secondly, as natural sources, they mainly derive from ruminant-derived foods (e.g., vaccenic acid (VA, C18:1, *trans*-11) and rumenic acid (RA, C18:2, *cis*-9, *trans*-11), VA is one of the most abundant TFAs in breast and cow’s milk and also dairy products. It is the intermediary precursor of RA, a conjugated linoleic acid (CLA) compound produced by bacterial biohydrogenation of FAs in the rumen of ruminant livestock like cows [14] (Figure 3). RA and VA in breastmilk originate mainly from dietary ruminant fat and the main source of ruminant fat in the diet is dairy fat, and to a smaller extent ruminant meat.

Clinical evidence indicates that natural TFAs have properties distinct from those of synthetically produced partially hydrogenated vegetable oils [14].

Wijga et al. [15] showed that a higher concentration of *trans* FA in breastmilk is associated with lower risk of atopic symptoms. Thijs et al. [16] confirmed these observations in children, by showing a protective association (with a plausible cause–effect relationship) of these FAs on the development of atopic manifestations. Chisaguano et al. [14] showed that higher maternal VA concentrations at week 12 of gestation were associated with a decreased risk of atopic eczema in one-year-old infants.

Mechanisms through which CLAs modulate immune function include influences on the production of cytokines, eicosanoids (prostaglandins, leukotrienes) and nitric oxide, inhibition of eosinophilic cationic protein, decreased availability of ARA or inhibition of either cyclooxygenase and mRNA expression of phospholipase A2, NFkappaB DNA-binding activity, and binding to PPAR-gamma [14].The EFSA Panel considers the current specifications for TFAs content in infant formulae and follow-on formulas as < 3 FAs % to be adequate [7].

## 3. Fats in Infant Formulae Intended for Medical Condition (According to the Regulation Eu n. 609/2013)

### 3.1. Preterm Formulae 

Lipids in preterm formulae provide the infant with the majority of energy, and the LC-PUFA (Table 1). In particular, the availability and metabolism of LC-PUFAs have direct implications for cell membrane functions and the formation of bioactive eicosanoids [17]. The supply of LC-PUFAs to the fetus during gestation is actively provided by maternal plasma. Thus, the preterm infant receives fewer LC-PUFAs prior to birth than a full-term infant and, therefore has a higher LC-PUFAs requirement at birth. Preterm babies lack of the so-called period of “biomagnification” when in late gestation the maternal supply of LC-PUFA progressively increases [18]. Accordingly, their daily needs per kilogram, or per 100 kcal, are greater in early life [19].

Preterm infant formulae are currently supplemented with commercially available sources of LC-PUFAs so that the fatty acid composition could be as similar as possible to human milk, but probably the supply of DHA and ARA should even be higher [19]. Most oils added to infant formulae are derived exclusively from microorganisms, others are derived both from a combination of low-EPA fish oil and microorganisms, sources of DHA and ARA, respectively. The usual DHA content of preterm formulae ranges between 0.2% and 0.4% of total FAs [20].

Since the coefficient of fat absorption decreases with the FAs chain length and increases with the number of double bonds, a high concentration of MCTs have been used in some preterm formulae to increase the coefficient of fat absorption in preterm infants [21]. The immaturity of digestive tract and high demand for energy further support the use of MCTs in preterm infants [21,22]. However, the MCTs content in preterm formulae should not exceed 40% of the total fats, [17] to avoid the increase of formula osmolality [23]. However, this is not broadly agreed. There is no evidence that either a deficiency or an excess of LA in preterm formulae is associated with adverse effects. However, LA intake between 385 and 1540 mg^−1^ kg^−1^ day^−1^ or between 350 to 1400 mg/100 kcal (3.2–12.6 E %) is currently considered acceptable. Both AA and DHA should be included in preterm formulae, and oils containing significant amounts of EPA should be avoided to prevent interference with the growth-promoting effects of ARA [24]. Recommended intakes are 12–30 mg^−1^ kg^−1^ day^−1^ or 11–27 mg/100 kcal for DHA and 18–42 mg^−1^ kg^−1^ day^−1^ or 16–39 mg/100 kcal for AA. The ratio of AA to DHA should be in the range of 1.0–2.0 to 1 (*wt*/*wt*), and EPA supply should not exceed 30% of DHA supply [17]. LC-PUFAs, added from fish oils or from single-cell algae, are actually added as triglycerides to the fat blend of preterm formulae [20]. The addition of DHA and ARA as enriched PL from egg yolk is maybe too expensive, even if it was found effective in the 1990s for enriched term-infant formulae [25].

Therapeutic formulae for preterms contain around 50% energy from fat, out of which up to 55% is obtained from MCTs, which allows for an appropriate proportion of essential fatty acids (EFAs), too. The usual DHA content of preterm formulae ranges between 0.2% and 0.4% of total FA [26].

A beneficial effect of the LC-PUFA supplementation in preterm formulae on neurological functions has not been demonstrated by meta-analyses so far. This may be due to the high variability in study designs and to the inclusion in the studied cohorts of relatively mature and mildly preterm infants, who are likely less DHA-deficient than extremely premature babies [20].

Moreover, the amount of LC-PUFAs used in early studies was chosen to produce the same concentration of ARA and DHA of the breastmilk (i.e., 0.2–0.4% fatty acids). This may not be an appropriate approach for preterm infants since the amount of DHA provided by breastmilk is below the accretion rate in utero. Indeed, infants fed with these formulae have constantly exhibited a reduced DHA status at time of discharge of hospital or expected term [20]. Several studies report outcome data in preterm infants fed with milk containing a high DHA content of 0.5–1.7% of total FAs. The first study that examined the effect of providing DHA supplementation (0.50% of total fatty acids) for up to nine months after term, showed that DHA improved growth in the whole cohort of preterm infants and improved mental development in boys [27]. Three out of six reports of preterm infants fed preterm formulae supplemented with n-3, but not n-6 LC-PUFAs showed some indices of lower growth [20]. Since then, all trials have investigated the effects of n-3 LC-PUFAs supplementation in preterm formulae together with ARA supplementation and none has demonstrated a negative effect of supplementation on growth [20]. Hence, the benefit of ARA supplementation is debated and, in any case, restricted to term infants [3].

### 3.2. Short Bowel Syndrome 

Short bowel syndrome (SBS) is a subcategory of intestinal failure, which may result from large resection of jejunum or ileum, and is associated with significant malabsorption. This condition is characterized by the inability to maintain adequate protein-energy, fluid, electrolyte, or micronutrient retention with a conventionally accepted, balanced diet. The clinical manifestation of SBS is determined by different variables such as the residual length of the jejunum and ileum, ostomies and their location, the presence (or absence) of the ileocecal valve, the remaining functional and absorptive length of the colon, underlying pathology and possible complications. After resection, the remaining portion of the intestine attempts to compensate for the lower efficiency of fluid and nutrient absorption, evolving through hypertrophy and muscular hyperplasia. Enteral nutrition plays an important trophic role per se, since the intraluminal nutrients may stimulate the epithelial cells and the production of tropic hormones [29].

Other available strategies for the clinician include absorptive adaptive and motility strategies needed to achieve successful intestinal rehabilitation in SBS. Even if in most SBS infants, feeding strategies alone are not sufficient to overcome the constraints of a reduced intestinal surface area to meet nutrient requirements, elemental or semi-elemental formulae, and continuous drip feeding are described to be successful [30,31,32]. The composition of main formulae available in Italy is showed in Table 2.

The switch towards the enteral nutrition of infants with SBS may be advantageous while decreasing the provision of an adequate caloric supply, so the pros and cons should be carefully evaluated on a case-by-case basis. The addition of fats, in the form of either LCTs or MCTs, to formulae is more frequent than that of carbohydrates or proteins since effects on formula osmolality are minimal [31].

LCTs undergo bile-dependent hydrolysis in the proximal bowel. The ileal and jejunal chemoreceptors detect the presence of long-chain FAs (LCFAs) and stimulate the secretion of PYY (YY Peptide) and GLP2 (Glucagon-like peptide-2). The latter mediates the phenomenon of the ileal and jejunal brake, which consists of the slowing of the proximal intestinal transit. LCTs also stimulate CCK (CholeCystoKinin) release, slowing gastric emptying. These entero-endocrine signals are studied for their potential trophic benefits, as well. N-3 LC-PUFAs were also shown to have anti-inflammatory effects while improving splanchnic circulation and cholestasis in these patients [8]. In a randomized controlled trial, using an n-6 based source of LCTs with an n-3 supplement to maintain appropriate ratios, preliminary results show positive effects on growth, feeding tolerance, and fecal consistency [31].

The digestive and metabolic properties of MCFAs, already mentioned in the previous sections, may be highlighted in SBS patients, and therefore formulae containing 40–60% MCTs with remainder as LCTs appear to be not only well tolerated but indicated for nutritional rehabilitation and maintenance. For clinical applications, some investigators suggested that the type of fats to be administered should be based on the remaining bowel length and capacity, surgical history and the probability of fat malabsorption secondary also to the lack of adequate concentrations of luminal bile acids [31]. In these patients, MCTs improved total fat and energy absorption [33]. This might be explained by considering that the colon serves as a digestive organ for energy salvage (CHO metabolism and MCTs absorption) and production of trophic factors, such as SCFAS [8].

If intolerance to additional LCTs occurs, MCTs might be a valid alternative, although MCT oil, immiscible in feeds, has been associated with lipoid pneumonia. Accordingly, in infants with esophageal abnormalities, vomiting, and/or severe reflux disease, caution is needed [31].

In spite of significant advances in the management of SBS in infants, further efforts are needed to translate newer dietary manipulations into standards of practice for the SBS population and prevent the widespread poor nutrition status in this population.

### 3.3. Atopic Disorders

Formulae based on partial (Hypoallergenic—HA formulas) or extensively hydrolyzed proteins (eHF) (mostly cow’s milk proteins, casein and whey, or vegetable proteins, mostly rice and soy), amino acid formulae (AAF), and milk from other mammals represent the dietary options available for the treatment (or even prevention) of cow’s milk allergy (CMA) [34] (Table 3).

Recently, instead of total avoidance of the offending compounds, new proactive dietary habits have been developed. Since food allergy results when there is a breakdown or the oral “tolerance” mechanisms, which leads to inappropriate immune responses to ordinary food allergens, it has been hypothesized that some nutrients, such as EFAs (and even the offending allergens), could positively influence this process [35].

Within this context, the fat quality in the formula becomes critical, since eicosanoids are key players in the mechanism of atopic disorders. The modulation of the LA/ALA ratio and their LC derivatives may offer the opportunity to balance the synthesis of their pro-inflammatory and pro-allergic lipid products, the eicosanoids, and the DHA-derived mediators (resolvins and protectins) [36].

The fat-associated dietary modulation may have direct effects on the inflamed gastrointestinal tract of atopic and food-allergic infants [37]. Inflammation may be viewed as a byproduct of the metabolic activity of gut microbiota from evidence that SCFAs are altered in overweight or atopic children [38].

More recently, the SCFAs acetate, butyrate, and propionate have attracted interest as mediators of allergic inflammations. They are produced by gut microbes and are used as an energy source by intestinal epithelial cells; after absorption, they may be easily utilized by the liver to increase gluconeogenesis [39]. Fatty acids can also play a strategic role in influencing the sensory profile of formulae for infants with CMA. In clinical practice both eHF and AAF are frequently rejected by children because of the taste. The unpleasant flavor of hydrolysis byproducts has been related to the peptides released during proteolytic processes, but recent observations suggest that palatability may improve, for instance, by manipulating levels of lactose and some FAs [40]. Oral fat exposure initiates cephalic phase responses throughout and beyond the gastrointestinal (GI) tract. To date, documented effects include: gastric lipase secretion, modulated GI transit, pancreatic exocrine secretions, gut hormone release, mobilization of stored lipid from enterocytes, pancreatic endocrine secretion, and altered lipoprotein lipase activity. As a consequence, dietary fats may influence appetitive responses, food intake, and nutritional status as a whole [40].

## 4. Conclusions

Since dietary fats are not only a source of energy but might also impact on health outcomes, the lipid composition of infant formulae is fundamental, particularly in children with special requirements.

In particular, the different types of fat may be crucial for the nutrition of preterm, short bowel, and atopic infants. Accordingly, MCTs can help to cover energy needs when the normal absorptive capacity of the digestive tract is compromised, while TFAs and metabolites of n-3 LC-PUFAs with their anti-inflammatory properties can modulate the immune function. Finally, the modification of taste by different fat types is a further issue requiring careful attention to improve the acceptance of these special products and compliance with therapeutic schedules.

## Figures and Tables

**Table 1:** Lipid composition of the principal available infant formulae for preterms.

Formula	Fat (% En tot)	TFA (g)	Saturated (g)	MUFAs (g)	PUFAs (g)	MCTs (g)	LA (mg)	ALA (mg)	EPA (mg)	ARA (mg)	DHA (mg)
**Pre Nidina ospedale (100 mL)**	44.4	4	1.7			1.6	560	80		16	16
**Pre Nidina casa (100 mL 14.4% *w*/*v*)**	46.8	3.8	1				373.1	75.2		14.1	14.4
**Pre-Humana (100 mL 13.5% *w* /*v*)**	43.6	3.2	1.1	1.5	0.7						
**Humana 0 (100 mL 15.4% *w*/*v*)**	46.7	4	1.4	1.8	0.9		739	80		17.6	17.6
**Humana 0-VLB (100 mL 15.4% *w*/*v*)**	41.7	3.9	1.3	1.9	0.7	0.6	549	69		21.5	21.5
**Plasmon Pre-0 (100 mL)**	46	4.2				0.8				25.1	14.7
**Plasmon 0 (100 mL)**	44.4	3.9	1.6	1.4	0.9	0.6	770	100		23.2	13.5
**Mellin 0**	43.8	3.9				0.2			2.9	17.7	13.6
**Mellin 0 POST (100 mL al 15.5% *w*/*v*)**	48	4	1.7	1.7	0.7	0.8	513	71	2.5	17	13
**Formulat Pre-0 (100 ml)**	44.5	4.1	1.25	1.7	0.8		670	97		28	21.5
**Formulat 0 (100 mL)**	45	4.1	1.4	1.7	0.8		670	97		24.6	21.3
**Pre- Aptamil (100 mL 14.4% *w*/*v*)**	48	4	1.5	1.8	0.6	0,8	490	76.7	2.3	17.4	13.2
**Similac 24 (100 mL)**	48.8	4.4					568			8	8
**Miltina 0 (100 mL)**	48	4				1.1				8	8
**Miltina 0 post (100 mL)**	48	4				0.8			2.5	17	13
**Blemil Plus (100 mL 15.9% *w*/*v*)**	45.5	4.1				0.5	611	49		24.6	16.4
**Eveil formula 0 (100 mL)**	45	4.1				0.6				24	12
**Nutribén Pre (100 mL)**	48.3	4.3				0.4				20	20

TFA: *Trans* fatty acids; MUFAs: monounsaturated fatty acids; PUFAs: polyunsaturated fatty acid; MCTs: medium-chain triglycerides; LA: linoleic acid; ALA: alpha-linolenic acid; EPA: eicosapentaenoic acid; ARA: arachidonic acid; DHA: docosahexaenoic acid; Data in Tables adapted from De Curti M et al. [28].

**Table 2:** Lipid composition of the principal available infant formulae for SBS.

Formula	Fat (% En tot)	TFA Total (g)	Saturated (g)	MUFAs (g)	PUFAs (g)	LCTs (g)	MCTs (g)	Omega-3 (g)	LA (mg)	ALA (mg)	ɣ-LA (mg)	ARA (mg)	DHA (mg)	EPA (mg)
Elemental 028 extra *	36	3.5	1.35	1.6	0.45	2.275	1.225							
**Monogen (100 mL 16.8% *w*/*v*)**	26.5	2.2	1.9	0.4	1.3	0.35	1.85							
**Peptamen Junior PHGG (100 mL)**	32	3.6	2	0.5	1.1		1.5	0.16						
**Alfaré (100 mL 13.5% *w*/*v*)**	43	3.4	2.1				1.3		480	60	17		5.7	
**Neocate LCP (100 mL 13.8% *w*/*v*)**	46	3.4	1.2	1.3	0.66	3.26	0.136		579	57.8		11.3	11.3	
**Infatrini Peptisorb (100 mL 14% *w*/*v*)**	49	5.4	3.4				2.7		421	83.9		15.8	15.7	
**Nutrini Peptisorb (100 mL 15% *w*/*v*)**	35	3.9	2.2	0.5	1.2		1.8		1010	92.7				
**Humana Disanal (100 mL 14.5% *w*/*v*)**	29.5	2.1	0.8	0.9	0.4				374	53				
**Aptamil- pepti Junior (100 mL 12.8% *w*/*v*)**	47.7	3.5	1.9	0.81	0.6	0.02	1.71		486.5	94.85	0.35	7	7	1.4
**Pantolac 1 (100 mL 13.7% *w*/*v*)**	46.4	3.4	1.5	1.3	0.5				418	84		11	6.4	1.4
**Pantolac 2 (100 mL 13.8% *w*/*v*)**	40.3	3	1.4	0.517	0.05				7.9	4.4				
**Vivonex Pediatric (100 mL 22% *w*/*v*)**	30.4	2.7					1.8		382					

* Oral formula only; Data in Tables adapted from De Curti M et al. [28].

**Table 3:** Lipid composition of the principal available infant formulae for atopy.

Formula	Fat (% En tot)	TFA (g)	Saturated (g)	MUFAs (g)	PUFAs (g)	LCTs (g)	MCTs (g)	LA (mg)	ALA (mg)	ɣ-LA (mg)	ARA (mg)	EPA (mg)	DHA (mg)
**Mellin Polilat 1 (100 mL 13.6% *w*/*v*)**	47	3.5	1.6	1.3	0.6			455	83		12	2.3	11
**Mellin Polilat 2 (100 mL 14.4% *w*/*v*)**	41	3.1	1.4	1.2	0.5			411	75		9	1.9	9
**Nidina H.A 1 (100 mL 13.1% *w*/*v*)**	45.7	3.4	1					536	65		7.8		7.8
**Nidina H.A 2 (100 mL 13.6% *p*/*v*)**	41.6	3.1	0.8		0.6			466.6	57		5.7		5.7
**Althéra (100 mL 13.2% *w*/*v*)**	45.6	3.4	1.3					500	50		7.4		7.4
**Alfaré (100 mL 13.5% *w*/*v*)**	43	3.4					1.3	480	60				5.7
**Pregestemil Lipil (100 mL)**	50.3	3.8					2.1				23		11.5
**Neocate Spoon (100 g powder)**	36	18.8	6.9	7.5	3.5	18	0.8	3222	322				
**Neocate LCP (100 mL 13.8% *w*/*v*)**	46	3.4	1.2	1.3	0.66	3.3	0.1	579	57.8		11.3		11.3
**Neocate Junior (100 mL 21.1% *w*/*v*)**	42	4.6	2	1.6	0.84		1.61						
**Humana AT 1 (100 mL 14% *w*/*v*)**	46.9	3.6	1.4	1.4	0.7			595	52		13		13
**Humana AT 2 (100 mL 13.4% *w*/*v*)**	46.4	3.4	1.4	1.3	0.6			569	50	0.7	13		13
**Aptamil pepti 1 (100 mL 13.6% *w*/*v*)**	47	3.5	1.9	1.29	0.62	2.73	0.13	479.5	87.85	16.8	12	2.45	11
**Aptamil pepti 2 (100 mL 14.4% *w*/*v*)**	41	3.1	1.4	1.14	4.36	2.41	0.11	479.5	87.85	0.7	9	2.1	9

Data in Tables adapted from De Curti M et al. [28].
